# Narratives of Dreams and Waking Thoughts: Emotional Processing in Relation to the COVID-19 Pandemic

**DOI:** 10.3389/fpsyg.2021.745081

**Published:** 2021-11-02

**Authors:** Rachele Mariani, Alessandro Gennaro, Silvia Monaco, Michela Di Trani, Sergio Salvatore

**Affiliations:** Department of Dynamic and Clinical Psychology, and Health Studies, University of Sapienza, Rome, Italy

**Keywords:** dreams, linguistic features, referential process, COVID-19, affective salience

## Abstract

The Coronavirus-19 (COVID-19) pandemic posed a significant challenge to the physical, mental, and emotional well-being of each individual. It also brought the importance of daily emotional management for survival to the forefront of every human being. Our study aims to explore whether emotional processes perform different functions during waking thoughts and night dreams during the first lockdown in Italy. Utilizing Multiple Code Theory (MCT), our goal is to verify whether waking thoughts facilitate a functional disconnection in order to manage the trauma caused by COVID-19. Two online forms were distributed to random participants in the general population, presenting a total of 49 reports of night dreams (23 males; mean age 33.45 ds. 10.12; word mean 238.54 ds. 146.8) and 48 reports of waking thoughts (25 males; mean age 34.54 ds. 12.8; word mean M. 91 words ds. 23). The Referential Process linguistic measures and Affect Salience Index were utilized. It was found that Affect Salience is present in both dreams and in waking thoughts; however, Referential Activity was higher in dreams and Reflection and Affect words were higher in waking thoughts. Two different processes of emotional elaboration emerged. The results highlight the use of greater symbolization processes during dreams and a higher emotional distance in waking thoughts. These results confirm that during the nocturnal processes, there is greater contact with the processing of trauma, while during the diurnal processes, defensive strategies were activated to cope with and manage life via a moment of the defensive disruption of daily activities.

## Introduction

### A Traumatic Global Experience: Dreaming and the Narrative Process During COVID-19

The present research project aims to offer an analysis and understanding of affective processing processes during a very difficult moment of human existence, such as the experience of the COVID-19 pandemic, which involved every single inhabitant of the earth.

The processing of emotional trauma is a theme that has been extensively studied (Murray and Segal, 1994; Rachman, 2001), but to date, new questions have emerged regarding it. Recently, the spread of the COronaVIrus Disease 2019 (COVID-19) created a unique historical context (Viscuso and Mangiapane, 2020), which we can consider as a real collective event that we assume to be universal. The entire world population felt at risk of extinction (Diamandis, 2021) and each human being has had to emotionally and cognitively adapt to it (Daly and Robinson, 2021). The lockdown rules imposed the safest procedures possible at the time, such as the mandate to wear masks and the “stay at home” imposition. After 1 year, these protocols are the “new normal” (Rab et al., 2020), but this new reality has had a huge impact on social life (Beam and Kim, 2020), work conditions (Bonacini et al., 2021) and the emotions related to these changes (Li et al., 2020). Research immediately showed that the relationship between the COVID-19 pandemic and mental disorders (Castellini et al., 2021; Czeisler et al., 2021; Wang et al., 2021) increased, when compared to pre-pandemic times (Rossi et al., 2020). While many researchers have focused on the impact of COVID-19 on mental health, the intent of this work is to explore, given the same traumatic event, how emotional processing processes perform different functions during dreams and waking thoughts. We utilized the exploratory medium of writing to understand the different emotional processes that occur during the day and during the night. Historically, narratives are the most direct modality used to explore the cognitive and emotive reorganization of individuals (Polkinghorne, 1995). The theoretical models that analyze the narratives (Pennebaker, 2000; Solano, 2013; Bucci, 2021a) highlight how it is possible to understand the emotional organization and the structure of the person’s defenses during a particular moment in life. The autobiographical narrative capacity is closely linked to the use of defenses and the ability to stay in contact with the different emotions that the events of external and internal reality create in each of us (Boag, 2020). For example, studies on the process of psychotherapy have shown how the progressive lowering of the ego’s defenses allowed for a greater awareness of one’s own experiences (Kramer et al., 2020; De Roten et al., 2021). Most researchers used the analysis of narratives to capture the experience and the cognitive and emotional elaboration put in place to face and cope with the pandemic (De Leo and Trabucchi, 2020; Negri et al., 2020; Creese et al., 2021; Herbert et al., 2021). At the same time, many studies have focused on dreams, as carriers of a common experience and the unconscious elaborations which emerged (Iorio et al., 2020; MacKay and DeCicco, 2020; Mota et al., 2020; Pesonen et al., 2020). In our work, we have tried to combine and compare the two types of writing to investigate how mental processes are adaptive to highly stressful events trying to preserve the main tasks of daily life.

### Emotional Communication Processing and Affective Salience: Two Prospectives in Exploring the Language of Emotion

One theory of emotional processing that has explored the processes of integration and disconnection of traumatic experience is the Multiple Code Theory (MCT). This model poses that information is processed in parallel (McClelland et al., 1989) among three different systems, which organizes the experience in a global and raw way: (1) the subsymbolic coding system is characterized by experience gradients, and they manifest as sensations and feelings in all sensory modalities, as well as in physical and motoric experience; (2) the non-verbal symbolic coding system, which is delegated to representations, images, and metaphors; (3) and the symbolic verbal coding system, which concerns words. From the beginning of life, humans think and interact in subsymbolic and symbolic formats (Bucci, 2021b). Bucci (2021b) argues that a Referential Process (RP) connects these three types of processing systems by transforming information from one to another. The RP allows people to express their emotional experiences into words. The theory has also been updated to include new advancements in social neuroscience, such as research on mirror neuron networks, social cognition, and embodied communication (Goleman, 1998; Gallese, 2009; Borghi and Cimatti, 2010). The RP and emotional communication are connected to Damasio’s notion of dispositional representations (Damasio, 2010) that provide a neurobiological basis for the concept of emotional schema. These innate dispositional representations can be associated to the arousal/emotional activation and to a non-conscious state of the self where raw bodily aspects are totalizing. Different theoretical perspectives suggest that affect and emotions could be conceived of in terms of the basic processes laying at the core of the self (see the notions of core-self by Panksepp, 1998, 2005) and the proto-self by Damasio (1999, 2010). These basic processes represent the first, fast, and frugal modes through which the subject appraises the environmental patterns (Gigerenzer and Goldstein, 1996) to trigger and orient cognitive processing (Muramatsu and Hanoch, 2005). For example, Barrett et al. (2007) and Barrett (2017) theory of constructed emotions suggests that affect guides actions and constructs perceptions in the present. Emotions are not distinct entities; rather they result in a continuous process aimed to map out the demands of the environment and the set modes to address its variations (Gross and Barrett, 2011). It has been highlighted (Posner et al., 2005) that distinct neural pathways do not serve distinct emotions, but that two underlying neurophysiological dimensions are used to organize emotions: that of arousal (the extent to which an emotion is associated with an individual sensation of energy) and valence (the extent to which an emotion reflects a negative or positive state of mind; Barrett et al., 2007). In sum, in emotion processing, arousal and valence are classification dimensions which organize the basic process aimed to monitor each individuals’ global relationship to the world that we refer to as emotion (Barrett and Lindquist, 2008).

Emotional arousal could be acknowledged as the extent of activation experienced as a reaction to stimuli (Lang et al., 1999; Deckert et al., 2020). However, arousal represents an autonomous psychological concept associated with emotional states that is indexed by the activation of the sympathetic nervous system, the autonomic nervous system, or the endocrine system (Russell and Barrett, 1999; McGaugh, 2004). Accordingly, in their review, Storbeck and Clore (2008) highlight the role of the production of arousal to intensify perceptions, evaluations, and enhanced long-term memory for events. This concludes that while the valanced affective cues serve as information about value, the arousal dimension provides information about the urgency to intensify processing strategies.

In sum, in analogy to several psychoanalytic streams of thought (Klein, 1937; Bion, 1967; Green, 1977; Matte Blanco, 1981; Bornstein and Masling, 1998; Beebe and Lachmann, 2002), arousal and valence could be understood of in terms of the basic affective representations regulating and organizing mental functioning which, in turn, is organized into emotions in order to map out answers to environmental demands.

### Connecting/Disconnecting Emotional Experience to Language

Scherer (2005) posits that the episodes which constitute an emotional schema’s combinations, called “profiles of appraisal,” are made up of the activation of the schema’s affective core in various circumstances. The speakers or writers (in our research- writers) are often not directly aware of the components of the affective core that organize the emotional schema, and it is not often clear how they feel at the moment. Naming emotional labels or trying to explain feelings is often opposing to the processes at play when trying to understand emotional feeling, according to MCT (Bucci, 2021a). The RP involves expressing the parallel flow of multiple experiences in the discrete single channel of the verbal mode. This process is based on three specific major functions of connecting experience to words: Arousal, Symbolizing, and Reflection/Reorganizing. Arousal is concerned with the activation of content that is outside or within the boundaries of awareness. The Symbolizing function follows the arousing material that has been accumulating outside of awareness and takes it in to a more accessible form, linked to linguistic form. A specific experience occurs that symbolizes components of a certain emotional schema that the individual can vocally convey in the form of a story, a memory, or a dream. Then, after the schema has been experienced and portrayed as a narrative or played out in the relationship, fresh reflections on previous experiences might combine during Reflection/Reorganization. All people who tell or write about their own experiences have the power to activate the RP by going through all three processes.

Bucci and Maskit (2007) hypothesize that if the RP is disrupted (e.g., by conflict or trauma), or fails to develop adequately, the verbal and non-verbal systems within the schemas may be disconnected, thereby affecting the organization of the schemas, the regulation of emotional arousal, and the construction of emotional meanings. The idea of disconnection within the emotional schemas can be applied to both somatic and mental pathology (Di Trani et al., 2018; Negri et al., 2019; Mariani et al., 2020; Negri and Ongis, 2021). A specific pattern of disconnection is given by the high use of defenses, such as rationalizations or an abstract language (Renzi et al., 2020; Mariani and Hoffman, 2021; Negri and Ongis, 2021). Other research highlights how high levels of arousal and rationalization can activate a disconnection, which generates a disorganization phase (Bucci and Crisafulli, 2021).

### Aim of the Study

Given this collective traumatic event, our research aims to explore whether emotional processing mechanisms perform distinctive tasks when writing reports of waking thoughts vs. writing reports of night dreams during the first lockdown in Italy. Our goal was to explore whether the reports of waking thoughts showed a functional disconnection to handle the traumatic event caused by the COVID-19 pandemic, as per MCT (Bucci, 2021b). The dream writing process activates an integration of the RP in order to integrate and elaborate the traumatic experience of the pandemic during sleep. During the day, emotional elaboration is functionally deactivated to manage normal adaptive functions. More specifically, we hypothesized that:

(a) Reports of waking thoughts and night dreams will show the same levels of arousal;
(b) Reports of night dreams will present an integrated RP, showing higher Referential Activity, which is useful in elaborating the COVID-19 pandemic experience;
(c) Reports of waking thoughts will show higher cognitive and abstract functions to explain emotional activation;
(d) The arousal activation will present different relations to the symbolizing process in reports of waking thoughts and night dreams.

## Materials and Methods

### Participants

The sample was recruited from the general Italian population during the first week of the first lockdown. Each person was randomly assigned to one of two groups. Participants completed the informed consent and a sociodemographic questionnaire. One group was asked: “What did you dream about tonight? Write down your dreams freely during the lockdown.” A group of 49 individuals (23 males and 27 females) participated in this task. The mean age of participants in this group was 33.45 (*SD* = 10.12). Of these participants, 30% graduated college and 70% had one degree; 14% lived alone, 62% lived with family, and 24% with lived with a cohabitant; 40% described themselves as single and 60% reported to be in a relationship. The other group was asked: “How are you experiencing this period of lockdown? Freely write about your experience.” A group of 48 individuals (25 males, 23 females) participated in this task. Group members had a mean age of 34.54 (*SD* = 12.8). Of these participants, 27% graduated and 73% had degree; 19% lived alone, 52% lived with family and 29% lived with a cohabitant; 35% described themselves as single and 65% reported to be in a relationship. The dreams dataset had a mean word count of 238.54 (*SD* = 146.8) and the waking thoughts report word count was smaller (*M* = 9, *SD* = 23).

This study was carried out in accordance with the code of ethics of the World Medical Association (Declaration of Helsinki), and approved by the Ethics Committee of Department of Dynamic, Clinical and Health Psychology of the University “Sapienza.”

### Measures

#### Affective Salience Index

This computerized measure assesses for Arousal in written text. The affective salience index (ASI) is based on the view that affect is a global embodied source of meaning (Fornari, 1976; Salvatore and Freda, 2011; Valsiner, 2021)—namely, that patterns of activation of the whole body provide the subject with the experience of the world as a global entity. A preliminary version of ASI was used in a recent study to analyze the evolution of the meaning behind the characterization of dreams of a patient throughout the course of psychotherapy (Gennaro et al., 2020). In the context of that study, ASI proved successful in estimating the saturation of the affect-laden meanings in the patient’s dreams. In this study, ASI followed a meaningful, though non-linear trajectory, which was globally indicative of their progressive increase of the patient’s capacity to elaborate unconscious, affectively relevant areas of the mental landscape. Further studies conducted (Gennaro et al., 2020) which compared ASI to an individual’s physiological index of propensity to affective arousal (measured by Heart Rate Variability), transcript semantic complexity (measured as Semantic Entropy Index) and lexical syntactic complexity (measured through Flesch Vacca Index), proved ASI to be a reliable text-based index able to detect the affect intensity contribution of affective meanings to the whole semantic content of the text under investigation. Accordingly, we consider ASI as a proxy of affective intensity, not so much as the sum of single emotions, but as the structuring of co-activation of affect, paving the way for the expression of emotions (Berrios, 2019).

ASI is based on an automated procedure of textual implementations by the ACASM procedure (Salvatore et al., 2012, 2017) via T-Lab software. First, a Lexical Correspondence Analysis (LCA), based on the textual context^∗^lexical unit matrix allows to define a factorial space, where each of the factors maps a component of the meaning being activated within the whole textual corpus. Moreover, the LCA output provides the coordinate on the factorial space of each textual unit in which the textual corpus is segmented. The factorial coordinate of the i-th textual unit on the j-th factorial dimension is a measure of the degree of their association—the higher the coordinate, the higher the association is, therefore, the higher the contribution of the j-th factorial dimension is to the meaning of the i-th textual unit.

Factorial coordinates are used to compute the ASI. More specifically, the ASI is calculated for each textual unit, as its Euclidian distance from the origin of factorial space. It is worth highlighting that the Euclidian distance is computed by using the coordinates of the first two factorial dimensions only, as in equation (1). This is so because the ASI assumes that the first two dimensions are the computational equivalents of primary affective dimensions.


(1) ASI=tCF1⁢(t)2+CF2⁢(t)2

ASI_t_ stands for the Affective Saturation Index of the textual unit *t*. CF1_(__t)_ and CF1_(__t)_ stand for the factorial coordinates of the textual unit *t*, on the first and second factorial dimensions, respectively. From (1), it can be seen that ASI increases when one or both of the factorial coordinates increase. Accordingly, ASI can be interpreted as a measure of the magnitude of the contribution of the two first dimensions of the factorial space to the meaning of the textual unit—the higher the ASI, the greater the contribution. Therefore, ASI can be considered an index of the degree of saturation of the affective meanings comprising the textual unit.

#### Italian Weighted Referential Activity Dictionary

The Italian weighted referential activity dictionary (IWRAD) is a computerized measure (Mariani et al., 2013) in the Italian language which is able to detect the Symbolizing Phase. It contains a list of 9,596 frequently used Italian words, each assigned a weight between 0 and 1, with 0.5 as the neutral value. A high score represents a high level of RA, which corresponds to a high level of concreteness, specificity, clarity, and imagery in the speech sample. Part of the value of the IWRAD derives from its power to assess a particular linguistic style (rather than only focusing on content) and to represent the unintended aspects of emotional involvement (Maskit, 2021). Linguistic measures of the Referential Process can be processed by the Discourse Attribute Analysis Program (DAAP; Maskit, 2021). In this study, the Italian version, IDAAP (Italian Discourse Attribute Analysis Program) was utilized.

#### Italian Weighted Reflection and Reorganization List

The Italian weighted reflection and reorganization list (IWRRL) is a computerized measure of the Reflection/Reorganization process in the Italian language. It contains a list of 1,633 frequently used Italian words, each assigned a weight between 0 and 1, with 0.5 as the neutral value (Negri et al., 2018). A high score on this measure represents high competence in reorganization and reflection in speech, referring to the degree to which the speaker is trying to recognize and understand the emotional significance of an event or set of events in their own or someone else’s life, or in a dream or fantasy. It is not about abstract reflection, but rather a person’s reasoning related to an experience that has been vividly experienced. Through IWRRL, it is possible to detect and model the reorganizing phase of the referential process. RR measures can be processed by DAAP (Maskit, 2021).

#### Italian Reflection Dictionary

The Italian reflection dictionary (IREF) is a test analysis list of content words concerning how people think and communicate their thoughts. The dictionary includes basic logic words and words referring to cognitive or logical functions or the failures of these functions. It is a measure of the abstraction-intellectualizing process. IREF measures can be processed by DAAP (Maskit, 2021).

#### Italian Affect Dictionary

The Italian affect dictionary (IAFF) is a list that contains 1,786 Italian words concerning how people feel and communicate feelings directly. It includes emotion labels, functions associated with affective arousal, and words indicating an emotional response, either positive or negative. Measures can be processed by DAAP (Maskit, 2021).

### Data Analysis

According to the hypotheses of the present work, in order to test for the different working processes of dreams and reports of waking thoughts in the context of traumatic experiences, a *t*-test comparison has been run involving the IDAAP linguistic measures, ASI, and the individuals’ elaborative capacity as independent variables. In order to explore the relationship between ASI and other Linguistic measures in the two types of narratives, Pearson’s correlation will be applied in reports of night dreams and waking thoughts.

## Results

The initial results of the two sample groups for the *t*-tests involving age, gender, education, and marital status did not show any significant differences. Further, the Chi-Square analyses didn’t show any significant results either. Therefore, we could consider the two sample groups as similar. The *t*-test comparison displayed insightful results; significant differences have been highlighted for several variables. Following our hypothesis, no differences were detected in the ASI comparison between reports of dreams and reports of waking thoughts, as shown in Table 1. We also found a significantly higher level of IWRAD in dreams with a considerable effect size (1.06). Reports of waking thoughts further showed significantly higher levels of IAFF, IREF, and IWRRL.

**TABLE 1:** Comparison COVID-19 pandemic dreams and waking thoughts writing.

**Linguistic measures^a^**	**Dreams N. Segments 247**		**Waking thoughts N. Segments 75**		**T**	**p**	**Effect size**
	**M**	**SD**	**M**	**SD**			
IREF	0.03	0.02	0.04	0.03	–3.135	0.002*	–0.41
IWRAD	0.52	0.012	0.49	0.021	9.008	0.000*	1.06
IWRRL	0.54	0.01	0.55	0.023	–2.941	0.004*	–0.38
ASI	0.77	0.87	0.74	1.02	0.208	0.835	0.02

Independent t-test, *p < 0.01.

*^*a*^IREF, Italian Reflection Dictionary; IWRAD, Italian Weighted Referential Activity Dictionary; IWRRL, Italian Weighted Reflection and Reorganization List; ASI, Affective Salience Index.*

Additionally, Pearson’s correlation also showed some interesting results (see Table 2). While it was found that the ASI is negatively correlated to IWRAD and IWRRL in reports of dreams, there was no correlation in reports of waking thoughts.

**TABLE 2:** Pearson’s correlation in reports of dreams and waking thoughts.

	**ISAFF^a^**	**IREF**	**IWRAD**	**IWRRL**
Dreams				
^b^ASI (n. 247)	–0.093	0.030	−0.136*	−0.218**
Waking thoughts				
ASI (n.75)	–0.186	–0.084	0.020	–0.095

Pearson’s correlation two tails p < 0.01*; p < 0.001**.

*^*a*^ISAFF, Italian Dictionary of, respectively, Sum of Positive, Negative, Neutral Affects; IREF, Italian Reflection Dictionary; IWRAD, Italian Weighted Referential Activity Dictionary; IWRRL, Italian Weighted Reflection and Reorganization List.*

*^*b*^ASI, Affective Salience Index.*

## Discussion

Our work explored the different narrative modalities in relation to the same traumatic event caused by the COVID-19 pandemic through the writing of dreams and waking thoughts. Our hypotheses argued that different emotional processes take place in the writing of dreams and in the writing of waking thoughts, therefore expressing a different form of adaptation to the event manifested in reality. Specifically, we hypothesized that the ASI in the narrated texts would not have been different between the narratives of the day and the narratives of the night. ASI, as a measure of arousal activation should be the same, given that the pandemic was shocking for the entire Italian population. Our results confirm our hypothesis. However, although the level of emotional activation was different, we hypothesized that the dream narratives allowed for a greater ability to connect the lived experience to language. In fact, according to the MCT (Severino et al., 1989), dreams are one of the greatest processes of emotional and symbolic connection. Indeed, our results confirm that symbolization processes are significantly greater in dream scriptures. These results further confirm psychodynamic theories (Sands, 2010) which state that dream processes enable the ability to symbolize emotional schemas and to represent negative and anguish feelings. Another hypothesis of ours concerned the processes of reflection/reorganization of the emotional experience. We hypothesized that this process was more involved in the diurnal writings, as an attempt to explain the pandemic situation and find rational solutions to the experience. This hypothesis was also confirmed. During the day, the individual needs to adapt more functionally to reality and disconnect the emotional activation that would otherwise dominate the day as well. During the narratives of the waking thoughts, cognitive organization should prevail.

Our last hypothesis argued that different relationships existed between the arousal shown in the ASI measure and the other linguistic measures referred to the RP.

To verify this hypothesis, we analyzed the correlations between the different measures. We hypothesized that the correlations between the levels of arousal by ASI and the other linguistic measures should be different. Generally, a good level of the RP that allows for the connection between emotions and language implies a correlation between the processes of Symbolization and Reflection/Reorganization. In fact, this process emerges only in dreams, while no correlations emerge between the measures in waking thoughts, which is also supportive of our hypothesis.

## Conclusion

In conclusion, these results open up interesting theoretical speculations regarding the function of dreams. Further research should be carried out to confirm these findings in a more generalized way, concluding that dreams allow for a facilitation in the elaboration of traumatic emotional experiences. Our results support the findings of researchers who found that dreams could play a role in the reorganization of experience, particularly, in reference to memories, trauma, and the general ability of problem solving (Fosshage, 2007; Mariani et al., 2021). These results are in line with the studies on the psychotherapeutic process, which highlight how dream processing allows for the processing of traumatic experiences (Gennaro et al., 2020). Much research has been carried out on dreams in relation to the pandemic and the main findings highlight the central role that emotional processing plays in the dream function. Our results, albeit taken with caution due to the large sample size, show that the nocturnal and diurnal affective processes perform complex but diversified functions, useful for human adaptation and the survival of trauma (Borghi et al., 2021; Giovanardi and Spangler, 2021).

## Limitations

Despite the interesting results gained evidencing the different emotional processes that took place in the writing of dreams and in the writing of waking thought, limits need not be underestimated. The sample size is quite narrow and limited to a specific population. Nevertheless, this work could be read as preliminary evidence used to implement further studies, with similar research designs, to shed light on the specific mechanisms underpinning the workings of dreams. For example, the comparison of the narratives of dreams and waking thoughts in traumatic and non-traumatic contexts could help provide further insight to the mechanisms paving trauma elaboration, which could provide useful implications for clinical practice. A long-term follow-up study could be one relevant direction for future research. Finally, more psychological variables could be researched in further studies in order to explore how they would influence the emotional regulation of individuals.

## Data Availability Statement

The raw data supporting the conclusions of this article will be made available by the authors, without undue reservation.

## Ethics Statement

The studies involving human participants were reviewed and approved by Ethical Committee of Department of Dynamic and Clinical Psychology, and Health Studies. The patients/participants provided their written informed consent to participate in this study.

## Author Contributions

RM contributed to all the phases of the study. AG and SM participated in research design development, in results interpretation, and in writing and editing the manuscript. MD participated in results interpretation and in writing the manuscript. SS supervised and monitored the project. All authors contributed to the article and approved the submitted version.

## Conflict of Interest

The authors declare that the research was conducted in the absence of any commercial or financial relationships that could be construed as a potential conflict of interest.

## Publisher’s Note

All claims expressed in this article are solely those of the authors and do not necessarily represent those of their affiliated organizations, or those of the publisher, the editors and the reviewers. Any product that may be evaluated in this article, or claim that may be made by its manufacturer, is not guaranteed or endorsed by the publisher.
